# Evaluation of a national framework for rational use of medicines in Kazakhstan and its role in improving medicine use practices at the organizational and national levels

**DOI:** 10.1186/s12913-024-12172-9

**Published:** 2025-01-09

**Authors:** Gulzira Zhussupova, Ainur Aiypkhanova, Saule Zhaldybayeva, Dinara Satmbekova, Tamila Akhayeva, Sholpan Kaliyeva

**Affiliations:** 1 Center for the Development of Scientific and Research Activities, Astana Medical University, 47 Abay Ave, Astana, 010000 Kazakhstan; 2 “Sanat” National Education Development Science Center, 13, Dostyk St, Astana, 010000 Kazakhstan; 3 Ministry of Health, Republic of Kazakhstan, Mangilik El 8, Astana, 010000 Kazakhstan; 4 The Republican State Enterprise On the Right of Economic Management “National Research Center for Health Development Named After Salidat Kairbekova” of the Ministry of Health of the Republic of Kazakhstan, Mangilik El 20, Astana, 010000 Kazakhstan; 5 Al-Farabi Kazakh National University, Al-Farabi Avenue 71, Almaty, 050000 Kazakhstan; 6 Department of Fundamental Medicine, Faculty of Medicine and Healthcare, Higher School of Medicine, Al-Farabi Kazakh National University, Al-Farabi Avenue 71, Almaty, 050000 Kazakhstan; 7 Department of Clinical Pharmacology and Evidence-Based Medicine, NCJSC “Karaganda Medical University”, 40, Gogolya St, Karaganda, 100000 Kazakhstan

**Keywords:** Rational use of medicines, Kazakhstan, Indicators, Primary health care

## Abstract

**Background:**

Kazakhstan inherited the Semashko health system model, known for the centralized adoption of rules at the Ministry of Health (MoH) level that regulate the healthcare system. In 2019 MoH established a national framework with indicators aimed at collecting qualitative and quantitative data from healthcare organizations as part of their annual self-evaluation, and biannual external evaluation by the National Research Center for Health Development (NRCHD). The purpose of this study was to pilot the MoH framework on rational use of medicines and evaluate its effects on medicine use practices in health care organizations and at the national level.

**Materials and methods:**

This cross-sectional study was conducted from October to December 2019 at 22 state-owned primary health care organizations (polyclinics) serving adults and children in Astana, Kazakhstan. Data were collected by trained surveyors visiting each organization. Data were converted to numeric values to arrive at compliance scores for each organization and analyzed to assess each organization’s compliance with set indicators.

**Results:**

The evaluation showed the rational use of medicines was assessed as “excellent” (90% < compliant) in 2 organizations (9% of the sample), “good” (75–89% compliant) in 7 organizations (32%), and “satisfactory” (50–74% compliant) in 13 organizations (59%), with an average compliance rate of 71% across the sample. 22 organization-specific evaluation reports were developed by the evaluator (NRCHD) and sent to health care organizations for corrective actions. Evaluation data triggered two improvements at the national level: correction of the default setting for trade names to international nonproprietary names within the physician ordering feature of the national health information system for medicines, and adoption of a national policy that allowed the exchange of unused stocks of medicines between polyclinics.

**Conclusions:**

Using the national framework allowed the evaluator agency and healthcare organizations to identify organization-specific gaps and triggered improvements in the use of medicines.

## Background

When Kazakhstan gained independence in 1991, it inherited the Semashko health system model [1]. The Semashko model was based on the universal right to health care, where services were provided free-of-charge under a centrally planned economy [2]. Health service delivery was built on a multi-level system of state-owned health care providers. Starting with primary care providers, which in rural areas consisted of geographically dispersed feldsher-obstetric stations and nursing stations (served by physician assistants, midwives and nurses) that were organizationally tied to district hospitals, and polyclinics in urban settings (outpatient care facilities that housed general practitioners, pediatricians, specialist doctors, laboratory and diagnostic services), the system included referring complex medical cases to a regional hospital (tertiary care provider in each administrative region, owned by local governments), and, if needed, further to a specialized national institute (a specialized hospital owned by the Ministry of Health).

It is useful emphasize the centralized regulation of the healthcare system under this model. The central government represented by the Ministry of Health played and continues to play a dominant role in policymaking and rule-setting in health care, practiced through the issuance of detailed rules by the Health Minister. What should be the content of policies and procedures at the organizational level has been typically defined by the Ministry of Health of Kazakhstan (MoH) in its orders. The practice of writing and approving policies and procedures at the organizational level began in 2009, with the establishment of the national accreditation system for healthcare organizations, including those on the safety and management of medicines in healthcare organizations [3]. Despite this new practice of setting organization-specific policies and procedures, healthcare organization staff and clinicians in Kazakhstan are accustomed to relying on nationally adopted rules and frameworks to guide their daily work.

The Semashko model did not have an explicit focus on the rational use of medicines, but medicines management was an integral part of care delivery. Decision-making on medicine selection, procurement and ordering was the function of doctors, guided by a committee of doctors, and the role of pharmacists was limited to medicine storage, distribution and accounting. After gaining independence, Kazakhstan implemented a World Bank project titled “Health Sector Technology Transfer and Institutional Reform” [4], which included a component focusing on the rational medicines policy. As a result, the National Research Center for Health Development of MoH (NRCHD) opened a division aimed at educating the public and providers on clinically justified and safe use of medicines (initially called the Center for Rational Use of Medicines). Later renamed as Division of Medicines Policy Development, this entity was defined as the evaluator agency in the first MoH policy approving the national framework for rational use of medicines [5].

The need for such an entity arose from the practice of irrational use of medicines, including the ordering of medicines that lacked documented evidence of effectiveness and efficacy globally (certain medicines were used only in the post-Soviet countries and were not part of clinical guidelines coming from high-income countries), or were not recommended within national clinical guidelines, or were used when not indicated. A review of studies conducted in Kazakhstan between 2011 and 2024 revealed irrational use of medicines, such as simultaneous prescription of more than five medicines, one or more of which were not clinically necessary, referred as polypharmacy [6, 7]; excessive and incorrect use of antibacterial and injectable medicines (16.9% of all prescriptions at the outpatient level) [8, 9]; prescribing drugs by trade names (giving preference to certain brands which is against the MoH policies in Kazakhstan) [7]; pharmacies dispensing prescription drugs without a doctor's prescription (in one study, 32% of respondents noted their doctor did not prescribe the antibiotic they bought, 35% of respondents confessed of using antibiotics when they were not prescribed, and 29% of doctors complained that pharmacies dispensed antibiotics without a doctor's prescription) [9]; and the use of ineffective medicines (medicines with unproven clinical effectiveness and efficacy) [7].

This is a worldwide problem as reported by WHO: “more than half of all medicines are prescribed, dispensed or sold inappropriately, and that half of all patients fail to take them correctly”, with the overuse, underuse or misuse of medicines resulting in wastage of scarce resources [10]. Out-of-pocket payments represented 36% of total health expenditure in Kazakhstan in 2016, below the WHO-recommended adequate financial protection of at or below 20%, with out-of-pocket costs mostly spent on buying medicines [1, 11]. MoH justified the national introduction of mandatory social health insurance reform in 2020 in part by the need to decrease out-of-pocket costs of households on medicines – a key argument in support of the reform, demonstrating medicines were a central issue in such a major health financing reform. Despite the positive improvements in quantitative metrics: the observed decrease in the share of out-of-pocket payments from 35% in 2019 (pre-reform) to 28% in 2020 (post-reform) [11], and improved coverage of medicines (more medicines, both in volume and the list of names and types of medicines, are covered) [12], the qualitative aspects of the medicines provision remain an issue. Experts note the distribution of health financing and coverage with medicines is unequal across geographic locations, gaps in coverage remain, and more medicines need to be included into covered outpatient medicines list so that unnecessary inpatient care can be avoided [13–17].

World Health Organization (WHO) defined two interrelated strategic areas in its 2019–2023 roadmap, one of which called for ensuring quality, safety, and efficacy of health products and the second one on improving equitable access to health products, which could be accomplished through regulatory system strengthening among other activities [18]. Kazakhstan strengthened its regulatory system by setting up and funding national agencies that act as stakeholders in medicines management, and by adopting national regulations that support MoH efforts in ensuring that medicines are safe, effective, and accessible. For example, the NRCHD is a health policy think-tank and a health technology assessment agency, which also houses the Division of Medicines Policy Development [19]. Other agencies working with medicines include the National Center for Expertise of Medicines and Medical Products, which ensures registration and monitoring of medicines; and the Social Health Insurance Fund, which acts as the purchaser of health services and medicines guaranteed free of charge under the benefits packages, ensuring equitable access to medicines. The regulatory system is based on the Code of Health and the Health System, adopted by the President of Kazakhstan, which is an overarching, comprehensive health law that defines the roles and the responsibilities of stakeholders in the healthcare system working to ensure safe and rational use of medicines [20]. The next level of regulations are orders of the Minister of Healthcare, which are national policies that are mandatory for implementation. Healthcare organizations establish organizational policies and procedures but that is a more recent practice, since about 2010. Prior to 2010, organizational policies were limited to establishing committees, and approving certain decisions, but not describing processes in detail.

In the environment where providers are accustomed to seeking guidance from the MoH, organizations hesitate to develop their own tools to assess performance and to guide practice. At the same time, in a punitive system where the government was long known for punishing providers for non-compliance with its rules through fines or written notices, organizations try avoiding audits by government agencies. Therefore, it was natural for the central government body to deliver its guidance to health care organizations through the approval of a framework, assisted by its quasigovernmental agency NRCHD [21], which is not a state body and has no punitive powers.

The MoH rule also required the results of external evaluation be sent back to healthcare organizations for corrective actions, and improvement suggestions at the national level be submitted to the National Formulary Committee. The Formulary Committee is an advisory body of the Ministry of Health of Kazakhstan, consisting of at least eleven people representing various disciplines and stakeholders (government agencies, specialized care providers, pharmaceutical industry, non-governmental organizations with special knowledge in clinical pharmacology and evidence-based medicine). The purpose of the Formulary Commission is to develop recommendations for the MoH to improve access to, and the safety of medicines and medical devices. The MoH envisioned that the framework would be used to stimulate rational and safe use of medicines as a result of annual self-evaluation by a facility, and biannual external evaluation by NRCHD [5, 21].

The research question was to find out how useful the MoH framework was in evaluating rational use of medicines in health care organizations. It was also of interest to explore if such evaluation led to any improvements at the organizational and national levels. Therefore, the purpose of this study was to pilot the use of the MoH framework on the rational use of medicines by conducting an external evaluation at 22 primary healthcare organizations in Astana, Kazakhstan. Objectives included assessing their compliance against the nationally set indicators, identifying areas for improvement in these organizations based on collected data, and identifying areas for improvement at the national level.

The importance of research questions for solving scientific questions and practical issues in rational medicine use in Kazakhstan lies in WHO's priority to ensure the quality, safety, and effectiveness of medicines, where regulatory system strengthening is one of the recommended activities [18]. Kazakhstan strives to expand access to medicines and ensure their quality, safety, and effectiveness, as demonstrated in a series of reforms, such as establishing the national formulary committee, funding designated regulatory agencies, and setting relevant laws and policies. This framework is the first tool that allows systematic data collection at the healthcare organization level specifically on the rational use of medicines. Data collected through the framework could stimulate corrective actions at organizational and national levels. The value of this tool could be understood better if it is widely used in practice and if collected data results in desired improvements.

## Materials and methods

The medicine use evaluation was conducted using the framework approved by the MoH of Kazakhstan [5]. Formally called “Rules on conducting the evaluation of the rational use of medicines”, this framework established 28 identical indicators for hospitals and for outpatient organizations (#1-#28 in Table 1), with additional two indicators (#29–30 in Table 1) for outpatient organizations, totaling 30 indicators used in this study. These indicators were broken down as 11 structure indicators, 11 process indicators, and 8 outcome indicators as shown in Table 1. The indicators were aimed at collecting qualitative and quantitative data on the following components of medicines management: 1) organization’s formulary committee work; 2) planning and procurement of medicines; 3) monitoring the prescribing practices by doctors; 4) data collection on medication errors; 5) data collection on adverse drug reactions; 6) analysis of the rational use of financial resources.

**Table 1:** National Framework for Rational Use of Medicines: List of Indicators

#	Indicator Name	Indicator Type
1	Availability of a document describing the use and management of medicines in the healthcare organization	Structure
2	Availability of a Formulary Committee in the healthcare organization	
3	Formulary Committee composition and structure and the presence of a clinical pharmacologist	
4	The presence of a drug formulary in the organization	
5	Availability of a health information system that provides current and continuous access to patient care information (including medication orders)	
6	Availability of informed consent forms for patients who receive parenteral and high-risk medications	
7	Availability of a multidisciplinary team on antimicrobial use	
8	Availability of a team or a function that monitors the validity of medication orders	
9	Availability of a standard operating procedure that defines the list of high-risk medications and sets rules for storing and labeling high-risk medications (prescribes using a designated red sign)	
10	Availability of a system to document adverse drug reactions	
11	Availability of a system to document and monitor medication errors	
12	The frequency of drug formulary review in the organization	Process
13	The number of Formulary Committee meetings per year	
14	Medical staff has access to independent and reliable information about medicines	
15	Availability of the organization’s drug formulary and information on availability of medicines to medical staff	
16	Availability of justification to include medicines into the drug formulary, taking into account local morbidity	
17	The presence of an infection control system	
18	The functioning of the adverse reaction registration system	
19	The functioning of the medicine administration monitoring	
20	The functioning of the medication error monitoring system	
21	The functioning of monitoring system for rationality of medication orders	
22	Frequency of training on the rational use of medicines for medical staff	
23	Medicines are indicated by their international nonproprietary name in the organization’s drug formulary	Outcome
24	The organization’s drug formulary complies with the national medicinal formulary of Kazakhstan	
25	Healthcare organization’s medical staff knowledge assessment on the rational use of medicines	
26	Assessment of the use of medicines (ABC and VEN analyses)	
27	Medicine consumption analysis based on a method using the established daily dose and actual medicine consumption data	
28	Proportion of medication orders with proven clinical effectiveness and safety (medicine is part of the national drug formulary)	
29	Proportion of injectable medicine orders out of all orders per patient	
30	Proportion of antimicrobial medicine orders out of all orders per patient	

This cross-sectional study was conducted at 22 primary health organizations serving adults and children in Astana, Kazakhstan, funded by a grant from the Ministry of Health “Methodological support for healthcare reform” awarded to NRCHD. The Ministry of Health classified this study as exempt from full review by the National Ethics Committee of the Ministry of Health based on the study design and no imposed risk to patients whose medical records were reviewed. Informed consent from participants was not required. Staff of participating organizations were assured of the confidentiality of their responses. Organizations were selected based on their scope of services and type (state-owned polyclinics), and their participation was voluntary. Recruitment was made through official letters sent to healthcare organizations by the evaluator agency. Private healthcare organizations were not invited to participate due to grant stipulations. Each of the 22 sites (primary healthcare organizations) was assigned a code (HCO 1–22) for confidentiality.

At least two trained surveyors from the evaluator agency (clinical pharmacologists, pharmacists, and general practitioners by background) visited each facility for 2 full days between October and December 2019. Data were collected by reviewing medical records and other documentation (logbooks, meeting minutes, organizational policies and procedures), from in-person observation of processes, verbal staff interviews with responses jotted down by surveyors, and written staff surveys using anonymously completed questionnaires in paper form (the data collection guide for the written questionnaire is provided).

For each indicator at each organization, collected qualitative data were analyzed to assess the organization’s compliance. Although each surveyor might have had variations in how they interpreted various forms of qualitative data, surveyors followed an instruction to use the same types and sources of data designated for each indicator. The same evidence (data element) could be used in assessing compliance under more than one indicator since some indicators assessed closely related processes or structures. Verbal answers to open-ended questions and written staff responses to questionnaires had to be supplemented by surveyors’ review of additional evidence to assess true compliance. This was done so that the cultural tendency for staff to make their employer “look good” fearing retaliation or punishments was addressed by verification of their statements through additional evidence such as meeting minutes, training records, etc. A “yes” or “no” answer also had to be supplemented by documentation of compliance (examples of implementation).

Data collected for each indicator was converted into quantitative scores based on the following parameters. Compliance level against each indicator was assessed on a 2-point scale, where 2 points meant full compliance (if there was evidence of supporting documentation and due processes were in place when observed on site); 1 point was assigned for partial compliance where evidence was incomplete or scarce, or implementation was inconsistent; and 0 was assigned to mark non-compliance if there was no supporting documentation or no evidence of implementation. The total maximum number of points for all 30 indicators was 60 per organization, which corresponded with 100% compliance. Each organization’s total score is made by summing the score for each of the 30 indicators. Compliance ranges based on organizations’ total scores were defined as “Excellent” for 90–100% compliance, “Good” (75–89), “Satisfactory” (50–74%), and “Unsatisfactory” (below 50%) as shown in Table 2.

**Table 2:** Compliance assessment: total scores of organizations and corresponding percentages

Numeric score (0–60)	Percentage equivalent	Compliance range
54–60	90–100%	Excellent
45–53	75–89%	Good
30–44	50–74%	Satisfactory
0–29	< 50%	Unsatisfactory

Different pieces of evidence were not intended to be used as a stand-alone source for assessing the rational use of medicines. The organization’s total score reflected evidence collected from various sources – the written questionnaire responses by staff, notes taken by external evaluators on site, including from their observations, document review, and medical record review.

Statistical analysis of data was done in the GraphPad Prism program. The analysis utilized the univariate and multivariate analysis of variance. Assessment methods for the rational use of medicines were also described in a recently published article [22].

Data were analyzed and documented in aggregate form without disclosing organizations (coded organization numbers) in a report submitted to the Ministry of Health. Evaluation methodology and findings with organization-specific recommendations for improvement were sent by the evaluator agency to the participating healthcare organizations for review and action. In addition, resulting national level recommendations were given to relevant national agencies.

## Results

Evaluation data showed the following results:

- two organizations (9% of the sample) scored 55 (from the possible 60), corresponding to “Excellent” compliance range (lines #2, 16 in Table 3);
- seven (32%) organizations scored between 45 to 53 points, corresponding with “Good” compliance (see lines #5–7, 9, 14–15, 17 in Table 3);
- 13 (59%) organizations scored between 30–44 points, which meant “Satisfactory” compliance.

**Table 3:** Rational drug use scores by indicator type in 22 healthcare organizations in Astana

№	Organization (coded name)	Structure indicator score (N max = 22)	Process indicator score (N max = 22)	Outcome indicator score (N max = 16)	Total score (N max = 60)	Total percent % compliance
1	HCO-1	*15*	*13*	*8*	**36**	**60%**
2	HCO −2	*21*	*20*	*14*	**55**	**92%**
3	HCO −3	*14*	*12*	*11*	**37**	**62%**
4	HCO −4	*17*	*13*	*10*	**40**	**67%**
5	HCO −5	*20*	*16*	*13*	**49**	**82%**
6	HCO −6	*20*	*18*	*12*	**50**	**83%**
7	HCO −7	*17*	*18*	*10*	**45**	**75%**
8	HCO −8	*15*	*14*	*7*	**36**	**60%**
9	HCO −9	*20*	*13*	*12*	**45**	**75%**
10	HCO −10	*18*	*13*	*7*	**38**	**63%**
11	HCO −11	*17*	*13*	*8*	**38**	**63%**
12	HCO −12	*17*	*13*	*11*	**41**	**68%**
13	HCO −13	*16*	*13*	*9*	**38**	**63%**
14	HCO −14	*18*	*20*	*8*	**46**	**77%**
15	HCO −15	*16*	*15*	*14*	**45**	**75%**
16	HCO −16	*20*	*21*	*14*	**55**	**92%**
17	HCO −17	*20*	*18*	*12*	**50**	**83%**
18	HCO −18	*17*	*15*	*12*	**44**	**73%**
19	HCO −19	*16*	*16*	*12*	**44**	**73%**
20	HCO −20	*14*	*7*	*11*	**32**	**53%**
21	HCO −21	*14*	*11*	*11*	**36**	**60%**
22	HCO −22	*14*	*14*	*11*	**39**	**65%**
**Average score (compliance %) by indicator type**		17 (77%)	15 (68%)	11 (69%)	**43**	**71%**

No organization was deemed “Unsatisfactory” with less than 50% compliance.

Evaluation data from 22 healthcare organizations by indicator type showed the following:

- *structure* indicators had the highest compliance level at 77% compared to other indicator types, with an average of 17 points out of the maximum possible 22 (11 indicators with a maximum score of 2 each), which corresponds to “Good” compliance using the same percentage designations as in Table 2;
- *process* indicators had an average score of 15 out of 22, which meant 68% average compliance and corresponded with the “Satisfactory” range;
- *outcome* indicators yielded 69% compliance, also in the “Satisfactory” range, based on the average score of 11 out of 16 possible (8 indicators with a maximum score of 2 each).

**Fig. 1:**
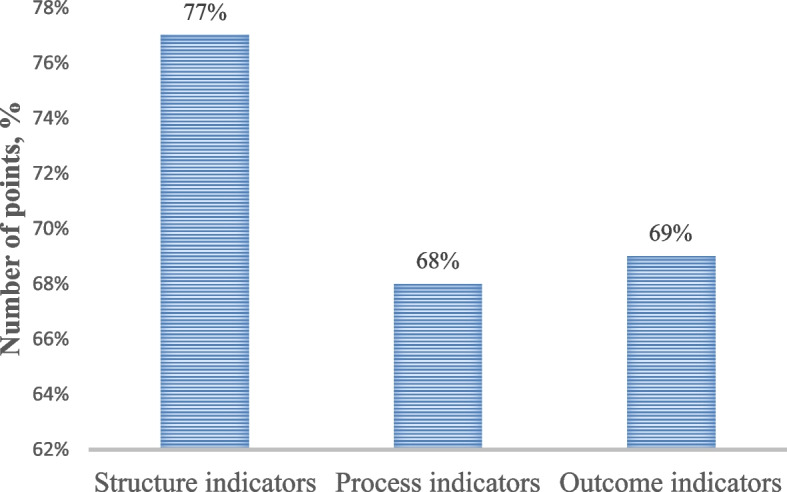
Average compliance across 22 organizations by indicator type. Note: Compliance by 22 healthcare organizations by indicator types. The average compliance for each indicator type is expressed in percentages. A statistically significant difference compared to the structure indicators is marked with an asterisk *, corresponding to *P* = 0,0404

The purpose of comparing compliance across the three indicator types was to see if structure indicators, that were more heavily affected by the national policies in Kazakhstan, had higher compliance than other types of indicators (see Table 3 and Fig. 1).

The evaluation revealed that the drug formularies of all healthcare organizations were developed correctly, had the desired standard structure, and all medicines were listed by international nonproprietary names (INN), as required by national regulations. However, when reviewing prescriptions and orders in medical records, 82% of orders in electronic records contained trade names only, without reference to INN. An error was detected in the national Information System for Medicine Provision, which resulted in doctors’ inability to select and prescribe all of the publicly covered medicines by trade name (not all trade names were included as an option in the information system), and no INN was referenced to each trade name. It was discovered during the evaluation, that when prescribing medicines using the INN, certain trade names were automatically displayed in the health information system which was part of the defect that needed correction.

Another system error related to the MoH-owned national information systems was revealed during data collection. Medical record review showed that 18% of medicines ordered were medicines not included in the Kazakhstan National Drug Formulary (KNF) [23] because at the time of data collection, the KNF list was not integrated with the Information System for Medicine Provision used by outpatient facilities to order and manage covered (state-paid) medicines.

One of the lowest compliances was with indicator #26 regarding the analysis of the use of medicines (ABC and VEN analyses). The majority – 19 organizations (86% of the sample) – did not analyze the rational use of financial resources for medicines.

Examples of organization-level improvement suggestions from the evaluator included additional training in areas of weak knowledge by staff (as evidenced by staff responses to the questionnaire); converting paper forms on adverse drug reactions to electronic format to integrate reporting with electronic health records; conducting microbiological tests before ordering antibiotics and implementing the list of reserved (restricted) antibiotics and others. The list of restricted antibiotics is a practice in Kazakhstan, required by national policies, where health care organizations create a list of antibiotics that require higher-level approval for ordering/prescribing (beyond just the treating doctor), due to their belonging to higher class and potency, high costs, and consequences for patient safety in case resistance to them is developed.

It was also discovered that prior to the evaluation, state-owned polyclinics had to follow a lengthy and bureaucratic procedure of acquiring a written permission from the government to exchange unused stocks of medicine when their needs changed. This practice led to the expiration of medicines and waste of resources by the time the written approval was received. The exchange was aimed at facilitating efficient resource management to allow a free-of-charge exchange of state-paid medicines among organizations so that those who needed the medicines could get them from those who no longer needed them. As light of this finding, the facilities expressed a desire to simplify and fasten the process.

## Discussion

The fact that two organizations (9% of the sample) scored high (55 points out of 60), suggests that the developed indicators can measure the intended parameters and can be applied to improving medication management in a healthcare organization. More than half (13/22, or 59%) of organizations scored between 30–44 of possible 60 points and had overall “Satisfactory” compliance (50–74% compliant), which meant that basic organizational processes and structures were in place. It is reassuring that none of the 22 public organizations were assessed as unsatisfactory, below 50% compliance.

The overall value of grouping indicators by type lies in resulting managerial actions, such as devoting resources to establish or better fund structures (for example, committees or organizational units), or spending time and effort on improving processes (reducing variations and improving individual staff members’ compliance), which in turn must lead to desired outcomes. However, the designation of each indicator as belonging to a certain type (structure, process, outcome) can be questionable because certain processes, structures and outcomes are very interrelated and become complex to detangle. Given that the current grouping of indicators has been approved as part of the MoH framework [5], it was of interest to find out if compliance across the three indicator types differed.

Authors predicted that in a Semashko-inspired healthcare model, largely retained in Kazakhstan, national policies guide much of what healthcare organizations have in terms of organizational units, their staffing, and committees. Therefore, it was more likely that structure indicators would have the highest compliance compared to other types, given that MoH policies were followed. This prediction was true as validated by the data shown in Fig. 1 and Table 3. Structure indicators had the highest compliance at 77% (17 points out of 22 on average), compared to 69% for outcome indicators and 68% for process indicators. This can be interpreted as organizations having established medication management systems based on uniform organizational structures, necessary committees, and having relevant organizational policies and procedures.

For example, the following structure indicators were likely implemented because the Ministry of Health enforced relevant national policies and created conditions that helped organizations comply with such expectations:

- to have an organization-wide program (document) describing the various stages of medication management and use;
- to have a Formulary Committee in the healthcare organization;
- to hire a clinical pharmacologist who should serve on the Formulary Committee;
- to have a drug formulary in the healthcare organization;
- to have a health information system for current and continuous access to patient care information;
- to have informed consent forms for high-risk medications (required as part of medical records);
- to have a system that documents and monitors medication errors and adverse drug reactions.

It is likely that organizational drug formularies’ compliance with the Kazakhstan National Drug Formulary (KNF) was influenced by relevant national regulations. Since 2016 Kazakhstan has introduced the KNF – a list of medicines with proven clinical safety and effectiveness, including medicines to treat rare diseases, which should be the basis for medicinal formularies of healthcare organizations. KNF is not limited to the WHO essential medicines list and provides access to a wider list of medicines with a description of reliable information about medicines at the knf.kz website [23].

Healthcare organizations could greatly benefit from ABC-VEN analysis, which allows judgment on the efficiency of spending on medicines. But the data showed that most (86%) of assessed organizations never analyzed the rational use of financial resources for medicines. The largest share of spending should be directed towards vital (V) and essential (E) medicines [24–27]. Not conducting such analyses is one of the most frequently found organizational-level gaps in this study.

The situation regarding the rational use and procurement of medicines was better in those organizations that had a part-time or a full-time clinical pharmacologist – a person who would advocate for effective and safe pharmacotherapy. There was no clinical pharmacologist in more than half – 13 (of 22), or 59% – of the organizations studied. Although it is natural that clinical pharmacologists are harder to be recruited and retained by an outpatient facility, MoH policies require at least a part-time person in such a role in polyclinics to ensure rational use of medicines, which in Kazakhstan are dispensed by polyclinics for all covered outpatient medicines, and not by retail pharmacies. In addition to managing and dispensing medicines included into the covered list, polyclinics have day-hospital units that administer medicines intravenously.

In Kazakhstan, a clinical pharmacologist is a medical doctor degree introduced since 2003, with a specialization in clinical pharmacology after receiving the medical university degree in medicine. In the absence of clinical pharmacologists, some facilities relied on pharmacists who do not receive any specialized post-graduate training after completing their bachelor’s degree and there are no PharmD equivalent degrees yet in the country. Pharmacists are trained mostly to manage medicines as goods (planning, procurement, supply chain management, storage, distribution across clinical units or dispending to patients) but their scope of practice excludes reviewing medicine orders or prescriptions alongside medical. Pharmacists can dispense a medicine according to an order but not question the order doctors – a practice since the Soviet times. Only in 2016 Kazakhstan’s MoH introduced a new degree titled “clinical pharmacist”, which is closer to the clinical pharmacologist in scope of practice, but the regulations are still incomplete in fully defining their functions and they are not yet widely known in practice.

It is noteworthy to mention that since 2009, Kazakhstan established the national accreditation system for health care organizations, which uses internationally recognized accreditation standards approved by the MoH and certified by the International Society for Quality in Healthcare (ISQua) [3]. As of 2021, 43% of healthcare organizations in the country complied with Kazakhstan’s voluntary accreditation standards that contained detailed requirements to medication management and use in accordance with evidence-based recommendations from high-income countries [28]. Since passing accreditation is voluntary, the organizations that chose to continuously improve patient care quality and safety are the champions driven by non-fiscal, non-regulatory motivations. The requirements for a health care organization’s medicines management system in the national accreditation standards and the MoH framework studied here are very similar, because they are derived from the same best international practices and recognized guidelines. Although the current share of accredited facilities is below 50%, the percentage of nationally accredited organizations is expected to increase over time, stimulating quality improvement and patient safety.

Thanks to the reliable increases in public funding, Kazakhstan kept up with equipping and supplying its facilities with the expected material resources, including medicines and medical equipment needed to safely store and distribute medicines, which are likely reflected in structure indicators. Data showed that overall systems for the rational use of medicines were in place as captured in structure indicators. However, it is harder to stimulate constant compliance with certain process and outcome indicators, that are up to the healthcare organization to enforce and self-audit.

Although the authors did not have a goal to ask for a feedback on this data collection exercise, and there was no question in data collection tools asking facilities whether they liked being evaluated like this, the authors received encouraging feedback from 15 out of 22 healthcare organizations about the value of participating in the evaluation. It was informal verbal and written feedback where staff reported they gained knowledge at no cost for them, got fresh insight to improve the use of medicines, and overcame the anxiety barrier of interacting with an external agency. Many said they were “expecting an audit-type interaction with the need to defend their practices” and had a fear of punishments following evaluation, but it turned out to be “a positive experience of peer-learning, brainstorming and solution-finding”. After detailed explanations from the evaluator agency on the purpose of the study, the organizations’ attitudes changed from being reluctant to participate to becoming eager to learn, ask questions, and seek advice.

Three system level findings were unexpected but were corrected afterwards with the involvement of relevant MoH agencies. One such finding was the defect with medication names in the Information System for Medicine Provision used by polyclinics to order, dispense, and account for state-covered medicines. The information system contained only certain trade names, but not all trade names, and lacked their linkage to the appropriate INN. When a doctor tried to prescribe by INN, a trade name had to be chosen from a list, which was an incomplete list. A standardized drug nomenclature is important because a medicine may be sold under different brand names, or a brand-name drug may contain more than one active substance. The use of INNs plays an important role in promoting the use of generic drugs, which improves drug availability and can reduce medication errors [29]. This error was corrected by relevant national agencies as trade names were linked to relevant INN, and prescription was made possible using INN and choosing the most suitable brand name. Organizations were reminded of the importance of using INN and the rationale to use less expensive brand names when their clinical efficacy met patient needs.

The second system error was rectified when the list of medicines in the Information System for Medicine Provision used by polyclinics was streamlined with the KNF list.

The third system improvement inspired by this study was the national policy adopted by the Ministry of Health allowing the exchange of remaining and unnecessary stocks of medicines among state-owned polyclinics. Medicines, including both injectable ones administered in the procedure rooms or day-hospital units of polyclinics, and outpatient prescriptions given to patients free of charge, are purchased and stored by polyclinics. If planning was inaccurate, the organization risks stocking piles of unwanted medicines, resulting in financial damage and waste of resources. This was found to be a significant issue that could be resolved if polyclinics were legally allowed to exchange medicines among each other. As a result, the evaluator agency initiated a change in the MoH policy, approved a year after this study, which enabled polyclinics to better meet the changing patient needs and balance inaccuracies in planning [30].

The framework was developed based on the best practices from high-income countries, but also considering the local context. For example, due to the local context, self-assessment tools do not yield reliable data in countries that historically had a punitive culture towards noncompliance, errors, or adverse events. Therefore, data collection in an environment where health workers are pressured to make their organizations “look good”, results in a need to collect data by external evaluators instead of relying on a self-assessment by a facility. External evaluations are costlier than self-assessments, and this factor limits the number of facilities that could be evaluated. Unless it is regarded as a voluntary self-evaluation tool, high data collection costs make the proposed framework difficult to implement nationally.

Limitations of this study included the following. Participating organizations were limited to state-owned outpatient organizations. Including hospitals would have provided a richer context where medication management is more complex, but it was not feasible given grant timelines and financial limitations. Not including private entities also leaves room for speculation on how practices would differ in the private sector where organization sizes are smaller, and the variety of staff is not as extensive as in state-owned polyclinics. Limited funding did not allow the participation of healthcare organizations from other regions of Kazakhstan. Studying only the capital city facilities may not reflect the situation in the entire country. The study also does not reflect the situation in the rural setting where multidisciplinary teams are difficult to establish. Finally, interpretation of facility performance may vary depending on the evaluator (surveyor bias) given the uncertainties with qualitative data interpretation and score assignment.

Based on available literature review in search engines (Web of science, Scopus), no studies to date were published on using a national framework for rational use of medicines in Kazakhstan, a post-Soviet country largely influenced by the Semashko model. The contribution of this study to scientific evidence is that for the first time, a national framework for rational use of medicines was tested in Kazakhstan. Collected data suggested basic processes and structures were in place, as recommended by ISQua-certified national accreditation standards for healthcare organizations, but some processes were not consistently enforced, such as laboratory-based justification for antimicrobial treatment. It was valuable to discover that lack of clinical staff knowledge in certain areas and shortage of clinical pharmacologists caused noncompliance with certain expectations set by MoH.

The value of this work may be enhanced if the MoH framework is validated and improved based on its further testing in an inpatient setting and across a larger sample size in Kazakhstan. When the government can stimulate improvements in patient safety and boost efficiency of resource use through regulations, it should take advantage of its role as the regulator. Given the historical context where healthcare organizations were guided by the MoH for processes, the MoH can maximize its positive impact on patient safety by adopting a framework like this. Although conditions for using the framework changed in 2020, when the Ministry of Health abolished the external evaluation part of it and designated the framework as a self-evaluation tool [31], it likely remains a useful guide for health care organizations in finding opportunities for improvement in the use of medicines.

## Conclusions

This study collected data from 22 primary healthcare organizations in Astana, Kazakhstan, to assess their compliance against 30 indicators included into the national framework for rational use of medicines adopted by the Ministry of Health in 2019. This was the first use of the framework in practice since its approval. Participating organizations acted in response to evaluation reports and made organization-specific improvements in the rational use of medicines. In 2020, the MoH changed its rule on the use of this framework, designating it as a voluntary self-evaluation tool and abolishing the external evaluation by NRCHD. Although the indicators in the framework and the approach to using it may evolve over time, the core idea of the framework – setting expectations for structures, processes, and outcomes in medicines management, and stimulating improvements is likely to remain valuable for health care organizations. Three national level opportunities for improvement were identified and implemented after the study was completed and included fixing discrepancies in electronic health information systems and simplifying medicine stock exchange among providers. This framework can be of value to other countries with a similar health system context.

## Data Availability

The datasets used and/or analysed during the current study are available from the corresponding author on reasonable request.

## Abbreviations

INN: International Nonproprietary Name
KNF: Kazakhstan national medicinal formulary
MoH: Ministry of Health of the Republic of Kazakhstan
NRCHD: National Research Center for Health Development
WHO: World Health Organization

## References

[1] OECD. OECD Reviews of Health Systems: Kazakhstan 2018. OECD Reviews of Health Systems, OECD Publishing, Paris. 2018. 10.1787/9789264289062-en.

[2] Krstic, K., Janicijevic, K., Timofeyev, Y., Arsentyev, E. V., Rosic, G., Bolevich, S., ... & Jakovljevic, M. B. (2019). Dynamics of health care financing and spending in Serbia in the XXI Century. Frontiers in public health, 7, 381 10.3389/fpubh.2019.00381. 10.3389/fpubh.2019.00381 PMC6927281 31921746

[3] Accreditation Center for Quality in Healthcare (2024). About us. Accreditation of Medical Organizations. https://acqh.kz/en/o-nas/ Accessed 21 Apr 2024.

[4] World Bank Group (2010). Health Sector Technology Transfer and Institutional Reform Project in Kazakhstan. https://documents.worldbank.org/en/publication/documents-reports/documentdetail/171131468292845249/kazakhstan-health-sector-technology-transfer-and-institutional-reform-project. Accessed 9 Dec 2024.

[5] Ministry of Health of the Republic of Kazakhstan (2019). “On approval of the Rules for evaluation of the rational use of medicines”. Order of the Minister of Health of the Republic of Kazakhstan dated May 6, 2019 No.-67. Registered in the Ministry of Justice of the Republic of Kazakhstan on May 8, 2019 No. 18636. https://adilet.zan.kz/rus/docs/V1900018636#z0.

[6] Nazarbayev A, Nurbakyt A, Omirbayeva B, Akhmetzhan A, Kosherbayeva L. Characteristics of High-Cost Beneficiaries of Prescription Drugs in Kazakhstan: A Cross-Sectional Study of Outpatient Data from 2022. Clinicoecon Outcomes Res. 2022;2024(16):827–37. 10.2147/CEOR.S470632. 10.2147/CEOR.S470632 PMC11572462 39559690

[7] Zhussupova G, Skvirskaya G, Esbatyrova L, Baidullayeva D, Kaliyeva Sh. A Review of the Drug Provision System for the Population of Kazakhstan and use of Medicines at the Outpatient Level. Current problems of health care and medical statistics, Issue. 2019;4:2019. 10.24411/2312-2935-2019-10100.

[8] Balapasheva AA, Smagulova GA, Mussina AZ, Ziganshina LE, Nurgaliyeva ZZ. Pharmacoepidemiological Analysis of Antibacterial Agents Used in a Provisional Hospital in Aktobe, Kazakhstan, in the Context of COVID-19: A Comparison with the Pre-Pandemic Period. Antibiotics. 2023;2023(12):1596. 10.3390/antibiotics12111596. 10.3390/antibiotics12111596 PMC10668668 37998798

[9] Zhussupova G., Makalkina L., Zhaldybayeva S., Zhetimkarinova G., Shakarovaa (2016). Current assessment of the antibiotics use by Public and Health workers. Kazakhstan Pharmacy. Astana, 2016. -№11 (186)-C.6–12.

[10] World Health Organization (2024). Promoting rational use of medicines, https://www.who.int/activities/promoting-rational-use-of-medicines, Accessed 7 Dec 2024.

[11] World Health Organization (2024). Health Expenditure Profile: Kazakhstan. https://apps.who.int/nha/database/country_profile/Index/en Accessed 9 Dec 2024.

[12] Ministry of Health of the Republic of Kazakhstan (2021). “On the approval of medicines provision under guaranteed volume of free medical care…” Order of the Minister of Health of the Republic of Kazakhstan dated August 20, 2021. No. ҚP ДCM-89/2021. https://adilet.zan.kz/rus/docs/V2100024069 Accessed 21 Apr 2024.

[13] Pichkhadze GS, E, Kushpeleva N,. Indicators Of Quality Drug Supply Within The Framework Of The Guaranteed Volume Of Free Medical Care In The Regions Of Kazakhstan. Value in Health. 2013;16(3):A248.

[14] Tukubaeva GN, Temireyeva KS, Serikbay AT. Drug provision of the Republic of Kazakhstan. Actual problems of our time. 2016;4:189–94.

[15] Kulzhanov MK, Zhanturiyev BM, Nadyrov PT, Kabdenova AT, Abdimanova BZh, Urazbaeva DCh, Datkhaev UM. The role of the National Center for Expertise of Medicines and Medical Products in drug provision of the population of Kazakhstan. Bulletin of the Kazakh National Medical University. 2019;1:644–6.

[16] Bukatov Y.B., Gimranova G.I., Shanin S.A. (2021). State Management of the Sphere of Circulation of Medicines in the Context of the COVID-19 Pandemic. Economy: strategy and practice. 2021;16(3):227–242. (In Russ.) 10.51176/1997-9967-2021-3-227-242.

[17] Zhussupova G., Aisina Zh., Makalkina L. (2015). Analysis of public drug supply on an outpatient level with in the guaranteed volume of free medical care using the funds of local budgets. Kazakhstan Pharmacy. Astana, 2016. -№11 (186)-C.13–18.

[18] World Health Organization (2019). Roadmap for access to medicines, vaccines and health product 2019–2023. Comprehensive support for access to medicines, vaccines and other health products. Geneva: World Health Organization; 2019. Licence: CC BY-NC-SA 3.0 IGO.

[19] Salidat Kairbekova National Research Center for Health Development (2024). History of RCHD. Accessed 1 Aug 2024 https://nrchd.kz/en/about-us/history-of-the-center.

[20] Tokayev K.K., President of the Republic of Kazakhstan (2020). Code of the Republic of Kazakhstan dated July 7, 2020 No. 360-VI ЗPК «On public health and healthcare system”. https://adilet.zan.kz/eng/docs/K2000000360 Accessed 21 Apr 2024.

[21] Zhussupova G.K., Zhaldybaeva S.S., Satmbekova D.K., Shakarova A.M. (2019). Assessment of the use of medicines in health care organisations. Methodological recommendations, 2019 - 82. https://drive.google.com/file/d/1WLfKZRGK7y5Q5PxzgS3fqwpSBjldseDc/view Accessed 1 Apr 2024.

[22] T.A.Akhaeva, L.M.Esbatyrova, S.S.Zhaldybaeva, G.K.Zhussupova, D.K. Satymbekova, A.M. Sejtalieva, A. Eleusizov (2023). Development of a methodology on the assessing the reasonability of prescribing medicines for assessing the rational use of medicines, Pharmacy of Razakhstan, №6 December 2023, 10.53511/PHARMKAZ.2024.98.71.020, http://pharmkaz.kz/wp-content/uploads/2024/02/6_2023-1.pdf Accessed 21 Apr 2024.

[23] Kazakhstan National Formulary of Medicines (2024). https://www.knf.kz/kk/site/index Accessed 21 Apr 2024.

[24] Yevstigneev SV, Titarenko AF, Abakumova TR, Alexandrova EG, Khaziakhmetova VN, Ziganshina LE. Towards the rational use of medicines. International Journal of Risk & Safety in Medicine. 2015;27(s1):S59–60. 10.3233/JRS-150690. 26639713 10.3233/JRS-150690

[25] Mohammed SA, Workneh BD. Critical Analysis of Pharmaceuticals Inventory Management Using the ABC-VEN Matrix in Dessie Referral Hospital. Ethiopia Integr Pharm Res Pract. 2020;2020(9):113–25. 10.2147/IPRP.S265438 PMC7490079 32983944

[26] 10.2147/IPRP.S265438.

[27] Mfizi E, Niragire F, Bizimana T, Mukanyangezi MarieFrançoise. Analysis of pharmaceutical inventory management based on ABC-VEN analysis in Rwanda: a case study of Nyamagabe district. Journal of Pharmaceutical Policy and Practice. 2023;16:1. 10.1186/s40545-023-00540-5. 36829254 10.1186/s40545-023-00540-5 PMC10129016

[28] Nguyen PH, Dang TVK, Nguyen PT, Vo TMH, Nguyen TTM (2022) 5-year Inventory management of drug products using ABC-VEN analysis in the pharmacy store of a specialized public hospital in Vietnam. Pharmacia 69(2): 517–525. 10.3897/pharmacia.69.e84348.

[29] Accreditation Center for Quality in Healthcare. Accreditation in numbers (2024). https://acqh.kz/ Accessed on Apr 2024.

[30] Gavrilova A, Zolovs M, Latkovskis G, Urtāne I. (2022). The Impact of International Nonproprietary Names Integration on Prescribing Reimbursement Medicines for Arterial Hypertension and Analysis of Medication Errors in Latvia. International Journal of Environmental Research and Public Health. 2022; 19(16):10156. 10.3390/ijerph191610156. 10.3390/ijerph191610156 PMC9408624 36011791

[31] Ministry of Health of the Republic of Kazakhstan (2020). “On approval of the rules for conducting an assessment of the rational use of medicines”. Order of the Minister of Health of the Republic of Kazakhstan dated November 3, 2020 No. ҚP ДCM-179/2020. https://adilet.zan.kz/rus/docs/V2000021586#z6 Accessed 16 Aug 2024.

